# Indoleamine 2,3-dioxygenase mediates immune-independent human tumor cell resistance to olaparib, gamma radiation, and cisplatin

**DOI:** 10.18632/oncotarget.1916

**Published:** 2014-04-18

**Authors:** Saman Maleki Vareki, Mateusz Rytelewski, Rene Figueredo, Di Chen, Peter J. Ferguson, Mark Vincent, Weiping Min, Xiufen Zheng, James Koropatnick

**Affiliations:** ^1^ Department of Microbiology and Immunology, Western University, London, Ontario, Canada; ^2^ Department of Pathology, Western University, London, Ontario, Canada; ^3^ Department of Oncology, Western University, London, Ontario, Canada; ^4^ Department of Physiology and Pharmacology, Western University, London, Ontario, Canada; ^5^ Cancer Research Laboratory Program, Lawson Health Research Institute, London, Ontario, Canada

**Keywords:** IDO- PARP- OLAPARIB- RADIATION- CISPLATIN- BRCA2

## Abstract

Indoleamine 2,3-dioxygenase-1 (IDO) is an immunosuppressive molecule expressed by most human tumors. IDO levels correlate with poor prognosis in cancer patients and IDO inhibitors are under investigation to enhance endogenous anticancer immunosurveillance. Little is known of immune-independent functions of IDO relevant to cancer therapy. We show, for the first time, that IDO mediates human tumor cell resistance to a PARP inhibitor (olaparib), gamma radiation, cisplatin, and combined treatment with olaparib and radiation, in the absence of immune cells. Antisense-mediated reduction of IDO, alone and (in a synthetic lethal approach) in combination with antisense to the DNA repair protein BRCA2 sensitizes human lung cancer cells to olaparib and cisplatin. Antisense reduction of IDO decreased NAD^+^ in human tumor cells. NAD^+^ is essential for PARP activity and these data suggest that IDO mediates treatment resistance independent of immunity and at least partially due to a previously unrecognized role for IDO in DNA repair. Furthermore, IDO levels correlated with accumulation of tumor cells in G_1_ and depletion of cells in G_2_/M of the cell cycle, suggesting that IDO effects on cell cycle may also modulate sensitivity to radiation and chemotherapeutic agents. IDO is a potentially valuable therapeutic target in cancer treatment, independent of immune function and in combination with other therapies.

## INTRODUCTION

Chemo- and radiation-resistance are major obstacles to treatment of most human tumors, regardless of their tissue of origin [1]. Identifying molecules underlying treatment resistance and targeting them therapeutically may sensitize tumor cells to chemotherapy and radiotherapy. Multiple mechanisms confer drug resistance on cancer cells, including alterations in drug metabolism, modifications to drug targets, dysregulation of apoptotic proteins, and enhanced DNA repair [2, 3] that can lead to clinical resistance to treatment [4]. The immunoregulatory molecule indoleamine 2,3-dioxygenase (IDO) is essential for oxidative catabolism of tryptophan in the kynurenine pathway [5]. Depletion of tryptophan and production of kynurenine compounds suppresses T cell activation and proliferation [5]. IDO in human tumors can facilitate immune evasion and tumor metastasis and lead to poor patient outcome [6-8]. In addition, other cells that reside in the tumor microenvironment, including dendritic cells and myeloid-derived suppressor cells (MDSCs), often express IDO and help to create tumor tolerance to tumor-infiltrating cytotoxic T cells [9, 10]. Notably, IDO induces T_reg_ cells to further suppress tumor-reactive T cells [11].

IDO appears to have additional, non-enzymatic functions, including a role in TGF beta-induced tolerance in plasmacytoid dendritic cells [12]. Moreover, the therapeutic activity of the BCR-Abl inhibitor imatinib has been attributed to IDO suppression and its consequent effect on T_reg_ cell function [13]. In contrast, it has been speculated that IDO can directly affect tumor cells, independent of effects on T_reg_ cells and other cells of the immune system. Such effects include blocking mTOR activity, inducing autophagy, and increasing NAD^+^ levels [14, 15]. Regardless of that, the relationship between IDO and non-immune related resistance to cytotoxic drugs or radiation in human tumors has not been studied.

Poly (ADP-Ribose) Polymerase (PARP) proteins are involved in cellular responses to DNA damage, including DNA repair and damage-induced cell death [16]. Therefore, limiting DNA repair by treating cancer cells with PARP inhibitors results in increased DNA damage. Hence these agents are being investigated as cancer treatment strategies [16]. Olaparib (AZD2281) is among the best-studied PARP inhibitors in both pre-clinical studies and human clinical trials [17, 18]. BRCA1/2 mutations in ovarian tumor cells may induce sensitivity to olaparib and reversion of BRCA gene mutations can reduce that sensitivity [19], but mechanisms underlying development of olaparib resistance to a greater degree than that mediated by functional BRCA is poorly understood. Furthermore, phase III trials of olaparib in ovarian cancer were terminated due to lack of increased overall survival in spite of evidence of olaparib-induced increase in progression-free survival [20]. Because most human tumors express IDO *in vivo* [21, 22] we investigated whether IDO in human tumor cells affected their response to olaparib treatment in the absence of immune cells.

Radiation is a common component of lung cancer treatment strategies, often combined with surgery, chemotherapy, or both. Inhibition of PARP1 enhances sensitivity to radiation in various tumor types including those of lung, ovary, and prostate; PARP inhibition in conjunction with radiation treatment could be effective in these cancers [20, 23, 24]. We investigated the capacity of tumor cell IDO to inhibit the combined therapeutic effects of olaparib and radiation. We report that increased IDO in human lung and cervical adenocarcinoma tumor cells conferred resistance to combined treatment with these agents, and antisense-mediated reduction in IDO sensitized cells to these treatments.

Radiation-induced DNA breaks in mammalian cells are normally accompanied by depletion of nicotinamide adenine dinucleotide (NAD^+^), a consequence that can affect the capacity of cancer cells to repair those breaks [25]. However, the effect of IDO-mediated NAD^+^ production on cancer cell sensitivity to radiation is not known. We show, for the first time, that IDO expression in cancer cells, independent of the immune system, conferred resistance to both olaparib and gamma radiation, alone and in combination with each other. Both gamma radiation and cisplatin can induce DNA double strand breaks (DDSBs) in cancer cells. Therefore, we examined the role of IDO in sensitivity to cisplatin. Inhibiting DNA repair by targeting BRCA2 is an attractive approach to sensitize cancer cells to chemotherapy [26]. We therefore combined IDO and BRCA2 downregulation is the context of cisplatin treatment. We report that antisense-mediated reduction of IDO in cancer cells sensitized those cells to cisplatin, alone and in combination with BRCA2 siRNA downregulation.

## RESULTS

### Generation of A549, HeLa and H441 clonal populations with high and low IDO expression

**Figure 1:**
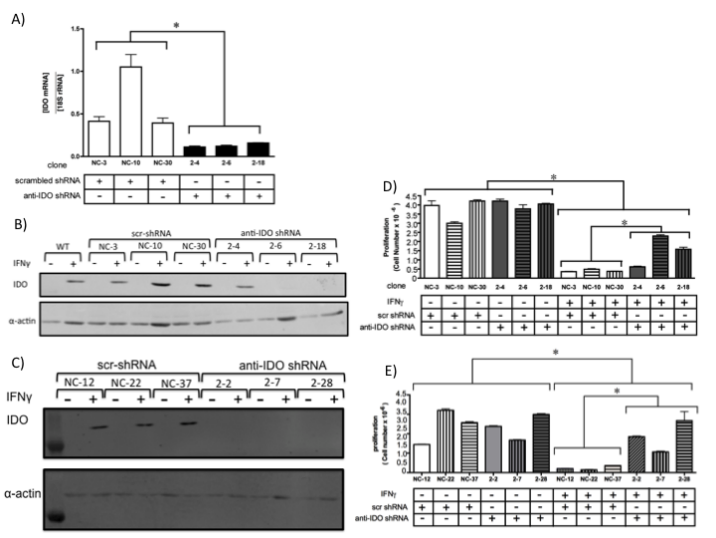
(A) IDO mRNA levels in A549 clonal cell populations 24 h after addition of IFN gamma to induce IDO Bars indicate the means of 2 independent measurements (*n*=3 for each measurement) ± SD (*, *p* ≤ 0.05). (B) IDO protein levels in individual A549 clonal populations, with and without IFN gamma treatment (48 hours). (C) IDO protein levels in individual HeLa clonal populations, with and without IFN gamma treatment (48 hours). (D) Proliferation of A549 clonal populations harboring anti-IDO shRNA or scrambled control shRNA, treated or untreated with IFN gamma (25 ng/ml, 72 h). Bars indicate the mean of 3 independent measurements ± SD. (*p≤0.05). (E) Proliferation of HeLa clonal populations harboring anti-IDO shRNA or scrambled control shRNA, treated or untreated with IFN gamma (25 ng/ml, 72 h). Bars indicate the mean of 3 independent measurements ± SD. (*p≤0.05).

We stably transfected human adenocarcinoma A549, HeLa, and H441 cells with plasmids directing production of anti-IDO shRNA or control non-targeting shRNA. Although most human tumors express IDO *in vivo* [27], IDO protein is undetectable in A549 and HeLa cells until induced by IFN gamma *in vitro*. Therefore, we used IFN gamma to induce IDO in both A549 and HeLa cells in these studies. However, H441 cells express IDO mRNA and protein without IFN gamma treatment *in vitro* (data not shown). Multiple A549, HeLa, and H441 clonal populations, with and without anti-IDO shRNA and with different basal levels of IDO mRNA and protein, were obtained (Figure 1, A-C, and Supplementary Figure 1). Upon IFN gamma stimulation, IDO-expressing clones (*i.e.*, those expressing control non-targeting shRNA) proliferated more slowly than IDO-downregulated clones (*i.e*, those expressing anti-IDO shRNA)(Figure 1, D-E). Because IDO expression is correlated with decreased proliferation [27] and the presence of anti-IDO shRNA attenuated IFN gamma-induced reduction in proliferation, these data suggest that IFN gamma-induced IDO protein is functional in these cells and that anti-IDO shRNA reduces IDO function.

### IDO in tumor cells affects cell cycle

**Figure 2:**
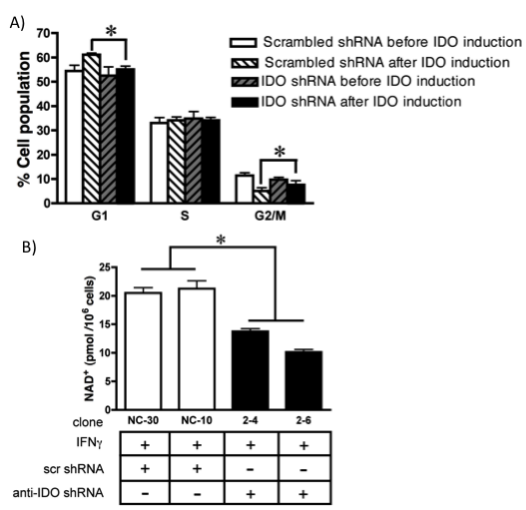
(A) Cell cycle analysis of A549 cells harboring anti-IDO shRNA or scrambled control shRNA, treated or untreated with IFN gamma (25 ng/ml, 48 h) Bars indicate the mean of 2 independently obtained measurements (*n*=3 for each experiment, total *n*=6) obtained from each of 3 clones harboring anti-IDO shRNA and 3 clones harboring scrambled control shRNA ± SD (*p ≤ 0.05). (B) NAD^+^ levels in A549-derived clonal cell lines (with and without anti-IDO shRNA) after induction of IDO for 48 h. The anti-IDO shRNA-transfected clones are statistically significant from the scrambled control shRNA clones. Bars indicate the mean of 2 independent measurements ± SD (*p≤0.05).

IDO slows proliferation in tumor cells [28] and IDO-mediated depletion of tryptophan induces cell cycle arrest in T cells at G_1_ [29]. We therefore determined whether IDO-induced reduction in growth of cancer cells was associated with altered cell cycle. IFN gamma induction of IDO increased the number of cells in G_1_ and decreased the numbers in G_2_/M in cells expressing scrambled control shRNA. The presence of anti-IDO shRNA in cells treated with IFN gamma abolished the increase and decrease, respectively (Figure 2A).

### IDO downregulation decreases intracellular NAD+

Because NAD^+^ is necessary for PARP activity [15], intracellular levels of NAD^+^ were compared in IDO-expressing and non-expressing cells. After IFN gamma stimulation, two independently-derived A549 clones expressing anti-IDO shRNA had lower amounts of NAD^+^ than two similarly-generated clones expressing scrambled control shRNA (Figure 2B). These data indicate that shRNA-mediated suppression of IFN gamma-induced IDO decreases intracellular NAD^+^ levels and has the potential to modulate PARP function.

### IDO in tumor cells mediates resistance to olaparib

**Figure 3:**
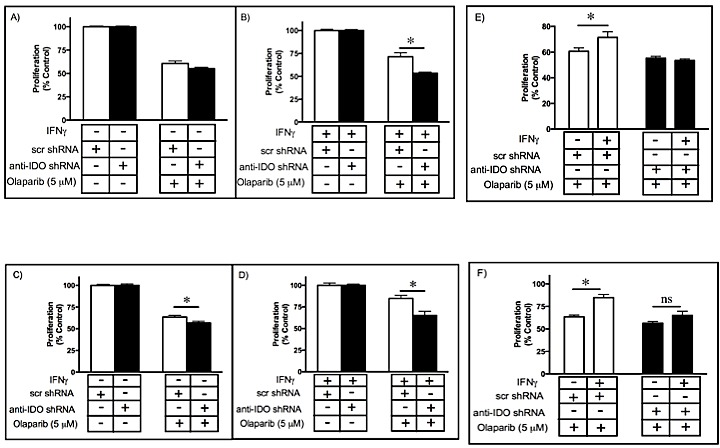
Sensitivity of clonal A549 populations to high dose olaparib (5 μM) before (A) and after (B) IDO induction. Results were obtained from 3 independent clonal cell populations harboring scrambled control shRNA or anti-IDO shRNA, and each bar represents the mean of the 3 values (*n*=3 for determination of each value) ± SEM (*, *P* ≤ 0.05). Sensitivity of clonal HeLa populations to olaparib (5 M) before (C) and after (D) IDO induction. Results were obtained from 3 independent clonal cell populations harboring scrambled control shRNA or anti-IDO shRNA, and each bar represents the mean of the 3 values (*n*=3 for determination of each value) ± SEM (*, *P* ≤ 0.05). Induction of IDO in A549 (E) and HeLa (F) clonal cell populations decreases the effectiveness of olaparib. Results were obtained from 3 independent clonal cell populations with scrambled control shRNA or anti-IDO shRNA, and each bar represents the mean of the 3 values (*n*=3 for determination of each value) ± SEM (*p≤0.05).

Several PARP inhibitors are undergoing clinical testing, alone and in combination with other treatments [20, 30]. We observed decreased levels of NAD^+^ (required by PARP) in human tumor cells after antisense-mediated reduction in IDO. We therefore hypothesized that IDO knockdown increases olaparib sensitivity. We induced IDO in A549 and HeLa clonal cell lines, with and without anti-IDO shRNA, by treatment with IFN gamma and then treated the cells with olaparib for 72 h. IDO downregulation in A549 cells increased tumor cell sensitivity to low dose (1 microM)(data not shown) and high dose (5 microM) olaparib by 18% (*P*=1×10^−3^)(Figure 3A and B and Supplementary Figure 2A). IDO downregulation sensitized HeLa cells to olaparib, but to a lesser degree than in A549 cells (Figure 3C and D and Supplementary Figure 2A and B). A549 and HeLa cells with unimpeded IDO expression after IFN gamma induction had increased resistance to olaparib, the effectiveness of the administered dose of the drug was reduced significantly, while antisense-downregulation of IDO during and after IFN gamma induction resulted in sensitivity to olaparib equal to that of cells untreated with IFN gamma (Figure 3E and F). These results show that IDO expression in tumor cells confers resistance to olaparib and, since all clonal populations were treated with IFN gamma identically, the observed resistance to olaparib was due solely to the presence of shRNA (and, by extension, IDO knockdown) and not effects of IFN gamma unrelated to IDO.

### Concurrent downregulation of IDO and BRCA2 further sensitizes A549 cells to olaparib

**Figure 4:**
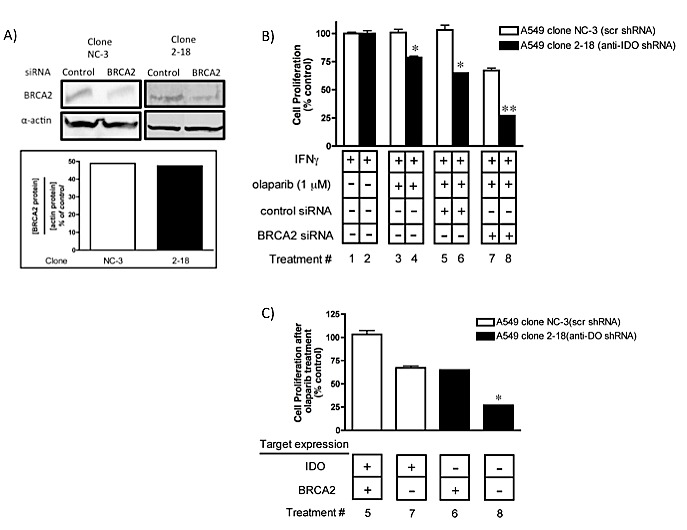
Concurrent downregulation of IDO and BRCA2 sensitized tumor cells to olaparib to a greater degree than knockdown of either target alone (A) BRCA2 protein downregulation after siRNA transfection. Bars represent the mean of two measurements. (B) Bars indicate the mean relative proliferation of cells from a representative experiment (n=3) ± SD. Each value was derived from data obtained from cells independently transfected with BRCA2 siRNA and treated with olaparib. Bars were normalized to values obtained from clones treated with IFN gamma but untreated with olaparib or siRNA; those cells were each considered to have proliferate at a 100% level after IFN gamma treatment. **Different from treatment with either siRNA alone (p≤0.05). (C) Data from panel B rearranged to show the effect of combined IDO and BRCA2 downregulation on A549 clonal population sensitivity to olaparib. *Different from values shown for lanes 6 and 7 (p≤0.05, Student's *t* test).

BRCA2 is important in homologous recombination repair (HRR). Tumor cells lacking BRCA1 and BRCA2 are sensitive to PARP inhibitors [31-33]. In view of the involvement of IDO in PARP-mediated DNA repair in cancer cells we hypothesized that concurrent downregulation of IDO and BRCA2 in cells would reduce PARP-mediated DNA repair and HRR, respectively, and sensitize cancer cells to olaparib more effectively than knockdown of either target alone. We transiently transfected A549 clonal populations (with and without stably-incorporated anti-IDO shRNA) with BRCA2 siRNA and then treated with olaparib. Concurrent downregulation of IDO and BRCA2 sensitized tumor cells to olaparib (1 microM) to a greater degree than after knockdown of either target alone (Figure 4), possibly by reducing the capacity of these cells to repair DNA lesions result of their genomic instability.

### IDO mediates resistance to gamma radiation in cancer cells

**Figure 5:**
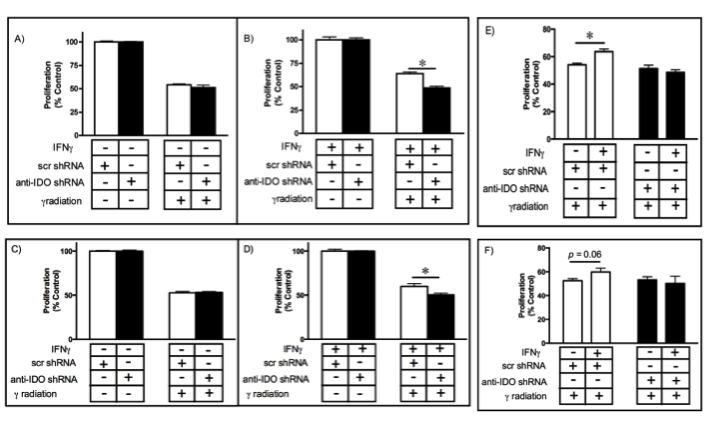
A549 (A-B) and HeLa (C-D) sensitivity to gamma irradiation (4 Gy) before (A and C) and after (B and D) IDO induction Results were obtained from independent measurements of proliferation of 3 A549 or HeLa clonal populations harboring control scrambled shRNA and 3 harboring anti-IDO shRNA. Bars represent the means of 3 independent measurements (*n*=3 for each measurement) ± SEM (*p≤0.05). IDO in A549 (E) and HeLa (F) clones decreases the effectiveness of gamma irradiation. Results were obtained from independent measures of proliferation of 3 A549 or HeLa clonal populations harboring control scrambled shRNA and 3 harboring anti-IDO shRNA. Bars represent the means of those 3 independent measurements (*n*=3 for each measurement) ± SEM. *Different from cells treated identically but without IFN gamma induction (p≤0.05, Student's t-test).

Radiation remains an important treatment modality for many solid tumors. Cancer cell capacity to repair DNA damage plays an important role in conferring resistance to radiation therapy, which exerts its effects by inducing DNA damage [34]. In view of the potential for IDO to modulate PARP activity, we hypothesized that human tumor cell IDO mediates resistance to gamma radiation. We induced IDO expression in A549 and HeLa clonal cell lines (with and without anti-IDO shRNA) with IFN gamma and treated with gamma radiation 48 h later. IDO downregulation sensitized cancer cells to radiation by approximately 20% (*P*=2.6×10^−7^)(Fig. 5A-D). Reduction in colony-forming capacity induced by gamma radiation was similarly enhanced by antisense reduction of IDO (*data not shown*). A549 and HeLa clones untreated with IFN gamma (*i.e.*, lacking IDO) were equally sensitive to radiation regardless of whether or not they harbored anti-IDO shRNA (Figure 5, A and C; Supplementary Figure 3). In addition, IDO induced by IFN gamma treatment of A549 clones lacking anti-IDO shRNA (*i.e.*, stably expressing only control scrambled shRNA) increased resistance to gamma radiation by approximately 15%, compared with no change in clones harboring anti-IDO shRNA (Figure 5E). HeLa cells showed a similar trend toward increased resistance after IFN gamma treatment, but not to a degree that achieved statistical significance (Figure 5F). These data reveal a previously unidentified role for IDO in mediating resistance to cancer therapy, independent of immune function but highly relevant to cancer treatment.

### IDO in human tumor cells mediates resistance to combined gamma radiation and PARP inhibition

**Figure 6:**
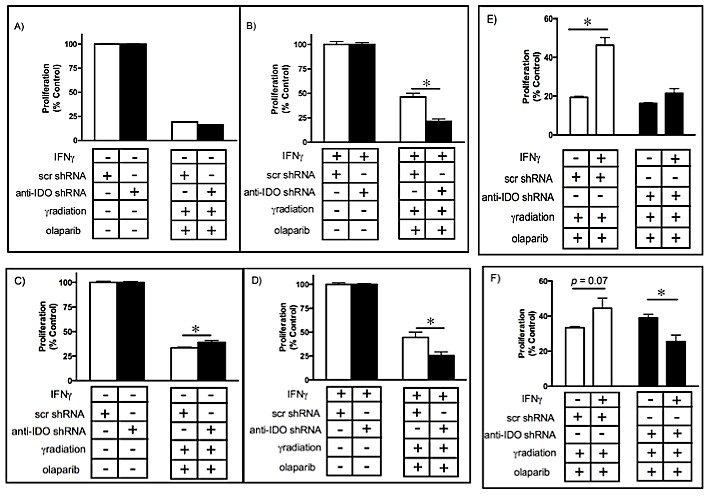
A549 (A-B) and HeLa (C-D) sensitivity to combined gamma irradiation (4 Gy) and olaparib (5 microM) treatment before (A and C) and after (B and D) IDO induction Results were obtained from independent measurements of proliferation of 3 A549 or HeLa clonal populations (2 independent experiments for each population) with control scrambled shRNA and 3 with anti-IDO shRNA. Bars represent the means of those 3 independent measurements (*n*=3 for each measurement) ± SEM (*p≤0.05). IDO in A549 (E) or HeLa (F) clones decreases the effectiveness of combined gamma irradiation and olaparib treatment. Bars represent the means of 3 independent measurements (*n*=3 for each measurement) SEM. *Different from cells treated identically but without IFN gamma induction (p≤0.05, Student's *t*-test).

In light of the common clinical use of combination therapies and the common goal of causing DNA damage and subsequently inhibiting DNA repair through the use of gamma radiation and PARP inhibitors, respectively, it was of interest to determine the effect of IDO on cell sensitivity to the combination of these treatments. We tested this hypothesis by inducing IDO in A549 and HeLa clones with IFN gamma and treating 48 h later with gamma radiation (4 Gy). Immediately after irradiation, cells were exposed to medium containing olaparib (5 microM) and assessed for proliferative capacity. Prior to treatment with IFN gamma, all clonal A549 and HeLa populations (harboring either anti-IDO shRNA or control scrambled shRNA) were equally sensitive to combined treatment (Figure 6A and C; Supplementary figure 4). After IDO induction with IFN gamma, A549 and HeLa clones harboring anti-IDO shRNA were approximately 56% and 20% more sensitive to combined treatment with gamma radiation and olaparib, respectively, than similarly-treated clones harboring control scrambled shRNA (p≤0.05)(Figure 6B and D; Supplementary Figure 4). In addition, IFN gamma induced IDO-mediated resistance to the antiproliferative effects of combined olaparib and gamma radiation, but anti-IDO shRNA abolished that resistance (Figure 6E and F). These data suggest that IDO counteracts combined treatment with gamma radiation and olaparib, and that antisense-mediated reduction of IDO in conjunction with combined treatments to induce DNA damage and inhibit DNA repair can be therapeutically beneficial independent of the role of IDO in immunity.

### IDO knockdown, alone or in combination with BRCA2 knockdown, sensitizes cancer cells to cisplatin

**Figure 7:**
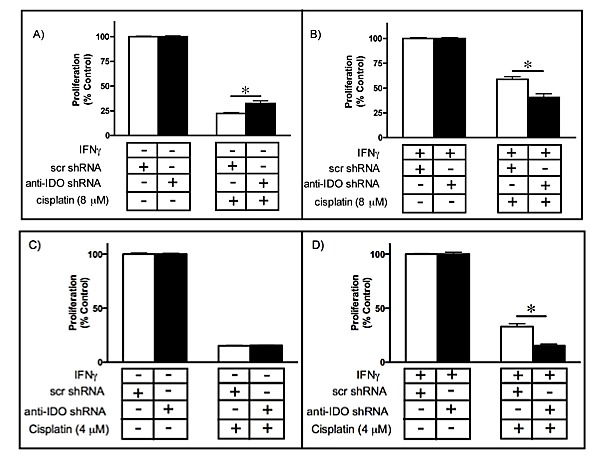
A549 or HeLa sensitivity to cisplatin (8 or 4 microM, respectively) before (A and C) and after (B and D) IDO induction Results were obtained from independent measurements of proliferation of 3 A549 or HeLa clonal populations harbouring control scrambled shRNA and 3 harboring anti-IDO shRNA. Bars represent the means of 3 independent measurements (*n*=3 for each measurement) ± SEM (*p≤0.05).

Since IDO downregulation sensitized cancer cells to gamma radiation, we determined whether IDO knockdown sensitizes A549, HeLa, and H441 cells to the DNA cross-linking agent cisplatin. We induced IDO in A549 and HeLa cells by treatment with IFN gamma but not H441 cells (natural IDO expressers) and then exposed cells to cisplatin to determine the effect on proliferation. IDO downregulation sensitized both A549 and HeLa cells to cisplatin treatment by 18% compared to cells without IDO reduction (p≤0.05)(Figure 7A-D). H441 cells stably transfected with anti-IDO shRNA were more sensitive to cisplatin than IDO-expressing H441 cells transfected with scrambled control shRNA (Supplementary Figure 5).

**Figure 8:**
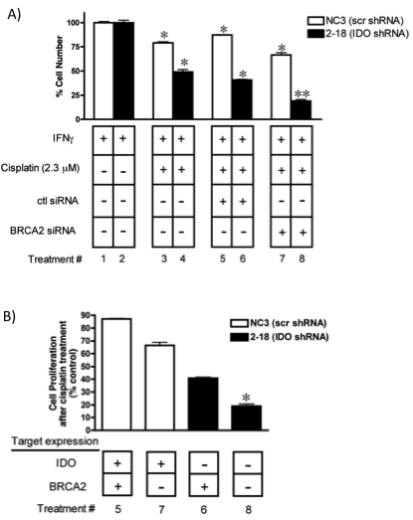
(A) Concurrent downregulation of IDO and BRCA2 sensitizes A549 to cisplatin in an additive fashion Bars represent the means of 3 independent measurements of cells (with or without downregulation of IDO) after BRCA2 siRNA transfection + cisplatin treatment (*n*=3 for each measurement) ± SEM. **Different from treatment with either siRNA in combination with cisplatin (p≤0.05). (B) Data from panel A rearranged to show the effect of combined IDO and BRCA2 downregulation on A549 sensitivity to cisplatin. *Different from values in lanes 6 and 7.

In addition, A549 clonal populations (with and without anti-IDO shRNA) were transfected with BRCA2 siRNA to inhibit DNA repair, treated with IFN gamma to induce IDO, and then exposed to cisplatin for 72 h to assess the effect on proliferation. Simultaneous knockdown of both IDO and BRCA2 sensitized A549 cells to cisplatin to a greater degree (70%) than either IDO knockdown alone (47%) or BRCA2 knockdown alone (20%)(Figure 8A-B). These data further suggest that blocking IDO in cancer cells in combination with cisplatin (and, potentially, other DNA-damaging cytotoxics), alone or in combination with drugs that target DNA repair (including potential antisense drugs) improve the efficacy of chemotherapy.

## DISCUSSION

IDO is expressed by most solid tumors (27), mediates tryptophan degradation, and plays a role in inhibiting immune cell cytotoxicity against tumor cells. Blocking the enzymatic function of IDO has been shown to sensitize tumor cells to chemotherapeutic anticancer drugs, but only in the context of an active immune system [35]. The data shown here reveal a new function for IDO, exclusive of immune activity, in mediating human tumor cell resistance to the PARP inhibitor olaparib, gamma radiation, and cisplatin. PARP proteins are essential for repair of single-strand DNA breaks (SSB), mainly through base excision repair (BER) but also in homologous recombination repair (HRR) and non-homologous end joining (NHEJ)[16]. Unrepaired SSBs can generate double strand breaks (DSBs) during DNA replication that can be fatal to cells. PARP inhibition is preferentially cytotoxic to cancer cells with BRCA1 and BRCA2 mutations [36], but that effectiveness is reduced in cells with intact BRCA function. The reduction in effectiveness is evident in the failure of olaparib to increase the overall survival of ovarian patients [19], suggesting that PARP inhibition alone requires improvement to confer sufficient therapeutic benefit. We found that IDO induced by IFN gamma decreased the effectiveness of olaparib in BRCA-normal lung adenocarcinoma A549 and cervical adenocarcinoma HeLa cells (Fig. 3E-F). Furthermore, antisense-mediated reduction in the amount of IDO induced by IFN gamma reduced cellular NAD^+^ in A549 human tumor cell line by approximately 60% (Fig. 2D), similar to the reduction observed *in vitro* after treatment with FK866 (a pharmacological inhibitor of NAD^+^)[37]. IDO catalyzes the rate-limiting step in tryptophan catabolism in the kynurenine pathway, which is a source of NAD^+^ in cells. Depletion of NAD^+^ by FK866 sensitizes cancer cells to apoptosis [38], and FK866 has been tested in a phase II clinical trial for treatment of chronic B-cell lymphocytic leukemia and cutaneous T-cell lymphoma [15]. IDO-mediated increase in NAD^+^ has the potential to counter the therapeutic effect of agents such as FK866 (unpublished data), particularly in combination with cytotoxic agents that induce genotoxic events (gamma radiation and cisplatin, for example). Increased NAD^+^ concomitant with IDO induction in human tumor cells may enhances PARP activity and resistance to PARP inhibitors (among multiple NAD^+^ roles [39] including NAD+-mediated resistance to reactive oxygen stress [40]). The capacity of IDO inhibition to concomitantly reduce the amount of NAD^+^ and enhance the effectiveness of PARP inhibitors suggests a causative link. Moreover, IDO inhibition by treatment with antisense or small molecule drugs including 1-methyl-tryptophan (1MT)[14], alone or in combination with FK866, could increase the therapeutic impact of NAD^+^ inhibition in tumor cells. We showed, for the first time, that IDO in cancer cells can diminish the therapeutic effect of a PARP inhibitor and that blocking IDO can increase the effectiveness of PARP inhibition.

We have recently reported that concurrent targeting of BRCA2 and thymidylate synthase in A549 and Hela cells with antisense molecules sensitizes these human tumor cells to cisplatin and melphalan in a fashion consistent with complementary lethality [26]. Combined antisense-mediated knockdown of IDO (with stably-incorporated shRNA) and BRCA2 (with transiently-transfected siRNA) increased sensitivity to either olaparib or cisplatin in an additive fashion (Figs. 4 and 8). These observations provide, for the first time, a rational basis for targeting IDO in the context of treatments that inhibit DNA repair and/or induce DNA damage in human tumor cells. Tumors harboring BRCA2 mutations are more responsive to treatment with alkylating agents such as cisplatin [41]. Most solid tumors, including ovarian and breast tumors, express IDO [27], suggesting that IDO inhibition (using either 1-MT or therapeutic antisense agents) in combination with DNA-damaging and/or DNA repair-inhibiting drugs will be of value.

Gamma radiation is commonly used to treat lung tumors, often in combination with chemotherapy [42]. However, cancer cell PARP activity is of utmost importance in supporting survival in response to radiation-induced DNA damage [34]. PARP1 inhibition enhances the sensitivity of lung tumor cells to radiation [43], and combined radiation and PARP inhibition is therefore an attractive treatment possibility. Yet most solid tumors express IDO, especially at the relatively advanced stages when therapeutic gamma radiation is used. The capacity of IDO to mediate resistance to gamma radiation (Fig. 5) could undermine the effectiveness of radiation alone or combined with PARP inhibition. Specific reduction of IDO increased the effectiveness of combined treatment with radiation and olaparib in an additive fashion (Fig. 6), suggesting that therapeutic IDO knockdown will be of value in enhancing combined treatments.

We have previously shown that IDO expressed by mouse tumors reduces the capacity of endogenous immune cells to recognize and kill tumor cells [8, 44]. IDO blockade in combination with passive or active immunotherapy has been proposed as a strategy to enhance immunotherapy effectiveness [45]. Furthermore, combining an IDO inhibitor (to enhance endogenous anti-tumor immune activity) with chemotherapy (to directly target tumors) has been demonstrated to be effective both *in vitro* and *in vivo* and dependent on a functioning immune system [35]. Our data similarly support the concept of blocking IDO in combination with gamma radiation or olaparib (alone and in together, and potentially with additional targeting of BRCA2 in an induced lethality strategy) or cisplatin, with the critical new information that IDO can potentiate the action of these treatment modalities independent of immune function and regardless of the capacity of endogenous immune cells to recognize and kill tumor cells. Moreover, most effector immune cells secrete IFN gamma as part of their anti-tumor cytotoxic function [6]. As we show in this study, IFN gamma-induced IDO in cancer cells can mediate tumor cell resistance to anticancer cytotoxic drugs and gamma radiation. Therefore, reducing IDO production and/or function using small molecule drugs or antisense molecules may be particularly valuable in to enhance combined treatment with cytotoxic drugs and active and passive immunotherapy approaches that depend on endogenous immune cell function.

IDO induction decreased tumor cell proliferation, increasing accumulation of cells in G_1_ and decreasing accumulation in G_2_/M. Antisense knockdown of IDO reduced accumulation of cells in G_1_ and partially restored proliferation (Fig. 2A). Increased time in G_1_ is important to increase the ability of tumor cells to undergo complete, error-free DNA repair capable of removing basal and therapy-induced DNA damage (30). The increase in the number of cells in G_1_ seen exclusively in IDO-expressing cell lines suggests a possible broader role for this protein in cell-cycle checkpoint control, allowing for repair of DNA damage during G_1_ phase of the cell cycle (18, 30). However, increased tumor cell proliferation in response to IDO targeting has the potential to be counterproductive due to the resulting increase in tumor growth [27]. However, IDO knockdown-mediated sensitization to olaparib, radiation, or BRCA2 knockdown (alone and in combination), and cisplatin, all abolished the pro-proliferative effect of IDO targeting and increased the anti-proliferative effect of those therapies. Regardless of IDO-mediated reduction in growth rate, tumor cells could benefit from IDO-mediated G_1_ arrest in the context of cytotoxic therapy; slowed cell cycle transit time can increase the amount and fidelity of DNA repair contributing to treatment resistance [46]. Tumor cells commonly have reduced G_1_ checkpoint function and depend preferentially on S and G_2_ checkpoints to avoid DNA damage-induced death [18]. IDO targeting as a strategy to enhance currently approved and novel induced lethality approaches can be valuable regardless of potential limitations associated with targeting IDO in the absence of concurrent therapies.

In summary, we show for the first time that IDO mediates immune-independent, tumor cell-autonomous resistance to the PARP inhibitor olaparib and gamma radiation (as single treatments and in combination) and cisplatin. Antisense knockdown of IDO increased human tumor cell sensitivity to olaparib, gamma radiation, and cisplatin. Therapeutic targeting of IDO appears to be of value, not only in increasing endogenous antitumor immunity, but also in enhancing tumor sensitivity to therapy independent of immunity.

## MATERIALS AND METHODS

### Cell culture

Human lung adenocarcinoma A549 cells, cervical adenocarcinoma HeLa, and lung papillary adenocarcinoma H441 cells were obtained from the American Type Culture Collection (ATCC), and maintained in minimal essential medium alpha (MEM alpha), Dulbecco's modified Eagle medium (DMEM) or RPMI-1640, respectively, supplemented with 10% (A549 and HeLa) or 20% (H441) fetal bovine serum (FBS), 100 units/ml penicillin and 100 μg streptomycin (pen/strep), hereafter referred to as growth medium.

### IDO downregulation

Human A549, HeLa, and H441 cells were stably transfected with short hairpin RNA (shRNA) antisense to human IDO1 (SuperArray, Mississauga, ON; using Lipofectamine 2000 (LFA2K) (Invitrogen, Burlington, ON, Canada) according to the manufacturer's instructions (see Supplementary Material, Supplementary Figure 1, and Supplementary Table 1).

### IDO mRNA quantitation

Stably-transfected A549 and H441 clonally-selected populations were collected 24 h after treatment with or without interferon gamma (IFN gamma)(25 ng/ml, R&D Systems, Minneapolis, MN), respectively. Cells were lysed (Trizol reagent, Invitrogen) and total RNA isolated according to the manufacturer's instructions. cDNA was synthesized by reverse transcription (MMLV-RT, Invitrogen) using 1 μg of purified RNA. IDO and 18s rRNA levels were measured simultaneously by multiplex real-time PCR amplification using a TaqMan IDO1 gene expression assay kit (Applied Biosystems, Carlsbad, CA).

### IDO protein detection and measurement

Cell extracts were prepared from A549 and HeLa cells cultured in 75 cm^2^ flasks and treated with IFN gamma (25 ng/ml). Cells were incubated for 48 h, washed twice with ice-cold PBS, harvested, and sonicated. Lysed cells were centrifuged at 15000 × RPM for 15 min at 4°C and the supernatant collected and stored at −80°C for future use. Protein extracts (20 microg) were quantified by BioRad protein assay, separated by electrophoresis through a 12% polyacrylamide gel, and then electro-transferred to a nitrocellulose membrane. Monoclonal antibodies against IDO (Abcam, Cambridge, UK) and actin (Sigma, St. Louis, MO) were used to detect and quantify these proteins on the membrane and were visualized using a Storm scanner (GE Healthcare Life Sciences).

### NAD+ quantification

NAD^+^ levels were measured in A549 clonal populations stably transfected with plasmids directing expression of anti-IDO shRNA or scrambled shRNA, using a NAD^+^/NADH quantification Kit (BioVision, Milpitas, CA; Catalog#K337-100). Briefly, 2 × 10^5^ cells were seeded into 25 cm^2^ flasks and grown overnight. Medium was replaced 16-24 h later with 3 ml of fresh growth medium containing IFN gamma (25 ng/ml). Cells were washed 48 h later with ice-cold PBS, pelleted by centrifugation, and extracted using 2 freeze/thaw cycles and NADH/NAD extraction buffer (400 microl). NADt (total NAD including NADH and NAD) was detected in 50 μl of extracted samples after addition of NAD cycling buffer and NAD cycling enzyme mix to a total volume of 100 μl. NADH levels were measured in a similar fashion in aliquots where NAD^+^ was degraded beforehand by heating the samples to 60 °C for 30 minutes. NAD^+^ levels were calculated by subtracting NADH levels from NADt levels. Samples were read at OD_450_ nm using a Wallac Victor^2^ plate reader (Perkin Elmer Life Sciences, Waltham, MA).

### Cell cycle analysis

A549 cells (2×10^5^) were cultured overnight and then treated with or without IFN gamma (25 ng/ml). Forty-eight h after IFN gamma addition, cells were washed with PBS, trypsinized and fixed in 70% ice-cold ethanol. Cells were washed with PBS 24 h after fixation and resuspended in 1 ml of propidium iodide (20 microg/ml)(Sigma Aldrich, St. Louis, MO) and 0.1% Triton X-100 (BDH Chemicals, Poole, UK) staining solution with RNAse A (Bioshop, Burlington, ON,Canada) for 15 minutes at 37°C. Cells were analyzed using a BD FACSCalibur flow cytometer (BD Biosciences, Franklin Lakes, NJ) and FlowJo software (Tree Star, Inc., Ashland, OR, USA).

### Olaparib and cisplatin treatment

A549 and HeLa cells (5×10^4^) were seeded into 25 cm^2^ flasks in 2 ml of growth medium, Medium was replaced with fresh growth medium with or without IFN gamma (25 ng/ml) 16-24 h after seeding. Twenty-four or 48 h after addition of IFN gamma, medium was replaced with fresh medium containing olaparib (1, 1.5 or 5 microM)(AZD2281, Selleckchem, Houston, TX) or cisplatin (2.3, 4, or 8 microM)(Sigma-Aldrich). Three days after addition of olaparib or cisplatin, cells were washed to remove the dead cells and particles and adherent cells were trypsinized and enumerated using a Coulter counter (Beckman, Mississauga, ON). Viability of the counted cells was confirmed by Trypan blue exclusion. H441 cells (5×10^4^) were seeded into 6-well plates in 3 ml of growth medium. Medium was replaced with fresh growth medium containing cisplatin (5 or 10 microM) 16-24 h after seeding. Seven days after addition of cisplatin, cells were washed to remove the dead cells and particles and adherent cells were trypsinized and enumerated by Coulter counting.

### gamma radiation treatment

A549 and HeLa cells (5×10^4^) were seeded into 25 cm^2^ flasks in 2 ml of growth medium. Culture media was replaced with medium with or without IFN gamma (25 ng/ml) 16-24 h later. Cells were exposed to gamma radiation (4 Gy) using a ^60^Co irradiator (London, Ontario, Canada) or a Varian Clinical 21EX Linear accelerator (Varian Medical System, Palo Alto, CA) using a 6 MV X ray beam (40 × 40 cm with 1.5 cm water equivalent buildup material) 48 h after addition of IFN gamma. After irradiation, medium was replaced with fresh growth medium without IFN gamma, and cells were allowed to proliferate for 72 h. Cells were then trypsinized and live cells were enumerated using a Coulter counter.

### Combined treatment with radiation and olaparib

A549 and HeLa cells (5×10^4^) were grown and irradiated as described above. Immediately after irradiation, medium was replaced with fresh medium containing olaparib (5 microM) and cells were allowed to proliferate in culture for 72 h. Cells were then trypsinized and live cells were enumerated using a Coulter counter.

### siRNA transfection

SiRNA transfection was performed as described previously [26]. Briefly, concentrations of siRNAs targeting human BRCA2 [OnTarget Plus SMARTPool BRCA^2^ (Dharmacon RNAi Technologies)] that reduced target mRNAs by approximately 70% by 24 h after transfection were determined (10 nM). BRCA2 siRNA (10 nM) and control non-targeting siRNA (2.5 nM) in serum-free MEM alpha and LFA2K (2.5 μg/ml) were incubated together for 20 min. The siRNA:LFA2K mix was then added to A549 cells that had been seeded, in triplicate, at 2×10^5^ cells per 25-cm^2^ flask 24 h beforehand. At 4 h after addition of siRNA:LFA2K, media was exchanged for fresh growth medium containing IFN gamma (25 ng/ml). Medium was replaced with fresh medium containing olaparib 16-24 h later. Tumor cell proliferation was enumerated 72 h later using a Coulter counter.

### Statistical analysis

Student's *t*-test (2-tailed) was used to determine differences between two means. One-way ANOVA was used to assess differences among multiple means. A *P* value of 0.05 was selected *a priori* to indicate a significant difference.

In some analyses, data were pooled from A549 clonal populations that expressed anti-IDO shRNA and compared to the pooled measurements of multiple clones expressing scrambled control shRNA.

## SUPPLEMENTARY FIGURES AND TABLES


