# Supplementary material for: Indoleamine 2,3-dioxygenase mediates immune-independent human tumor cell resistance to olaparib, gamma radiation, and cisplatin
Source: Oncotarget. 2014 Apr 18;5(9):2778–91. doi: 10.18632/oncotarget.1916 (PMC4058044; doi:10.18632/oncotarget.1916)
Supplement: Supplementary file 1 [file oncotarget-05-2778-s001.docx]

Indoleamine 2,3-dioxygenase mediates immune-independent human tumor cell resistance to olaparib, gamma radiation, and cisplatin

**Appendix A**

**Supplementary material:**

**IDO downregulation in human tumor cells**

A549, HeLa, or H441 cells (1 x 10^6^) were cultured overnight in T25 flasks in 2 ml of MEM alpha supplemented with 10% FBS. On the day of transfection with shRNA, cells were approximately 70% confluent. For transfection, 10 µg of anti-IDO gene-specific shRNA plasmid or the scrambled control shRNA plasmid was mixed with 10 µl LFA2K and 125 µl serum-free MEM alpha. The mixture was then incubated for 20 minutes in room temperature to allow shRNA: LFA2K complex formation. After incubation, 250 µl of the mixture was added to each flask of cells. At 4 h after transfection, media was exchanged for fresh MEM alpha containing 10% fetal bovine serum. Cells were washed with PBS (1x) and trypsinized 24 h later, and seeded into a 14 cm petri dish in 30 ml MEM alpha supplemented with 10% FBS. Cells were allowed to proliferate in culture for 72 h then medium was replaced with fresh medium containing 2 µg/ml puromycin (Bioshop, Burlington, ON). Medium was replaced every 3 days with fresh medium containing 2 µg/ml puromycin. Stably-transfected cells formed colonies, and single colonies (approximately 30 per shRNA group) were selected and grew in 48-well plates in 0.8 ml MEM alpha supplemented with 10% FBS and 2 µg/ml puromycin. When confluent, cells were transferred to 6-well plates and were cultured in triplicate. A549 and HeLa cells were then treated with IFNγ (25 ng/ml) and IDO mRNA and protein levels were measured by qPCR and immunoblotting, respectively. H441 cells were assessed by qPCR for IDO mRNA expression without IFNγ treatment.

**Supplementary Table.1: SureSilencing shRNA plasmid sequences. Each anti-IDO shRNA sequence targets a specific exon on IDO gene. These vectors are used to stably transfect cancer cells.**

| **shRNA ID** | **Insert Sequence** |
| --- | --- |
| 1 | AGACTGCAGTAAAGGATTCTT |
| 2 | GTGACTAAGTACATCCTGATT |
| 3 | CAGTGTTCTTCGCATATATTT |
| 4 | TCCTCCAGGACATGAGAAGAT |
| NC (control) | GGAATCTCATTCGATGCATAC |

**Supplementary Figures:**





**
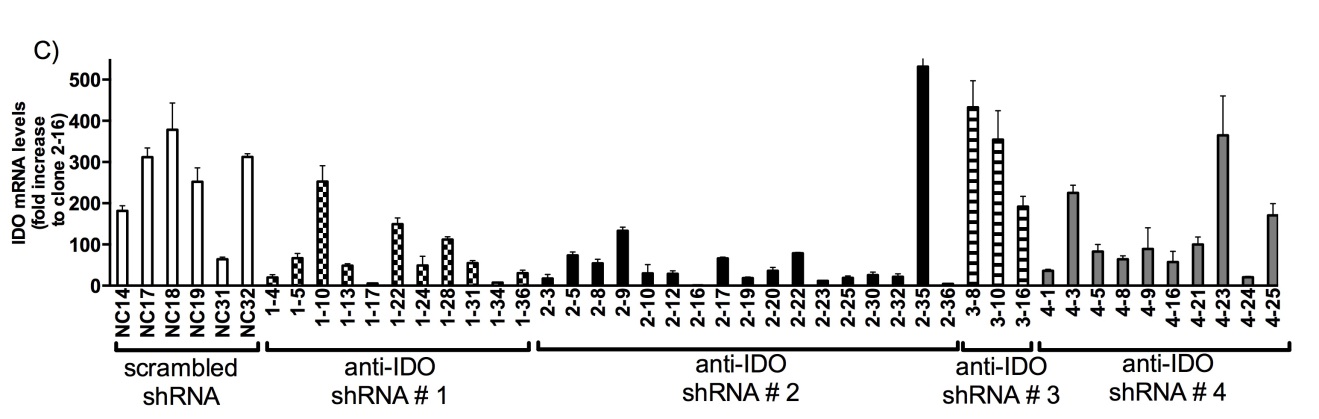
**

**Supplementary Figure 1:** IDO mRNA levels in A549 (A-B) and H441 (C) clonal cell populations 24 h after addition of IFNγ to induce IDO (A549) or culture medium only (H441). Clonal populations for examination were selected from among these candidate stably-transfected populations.


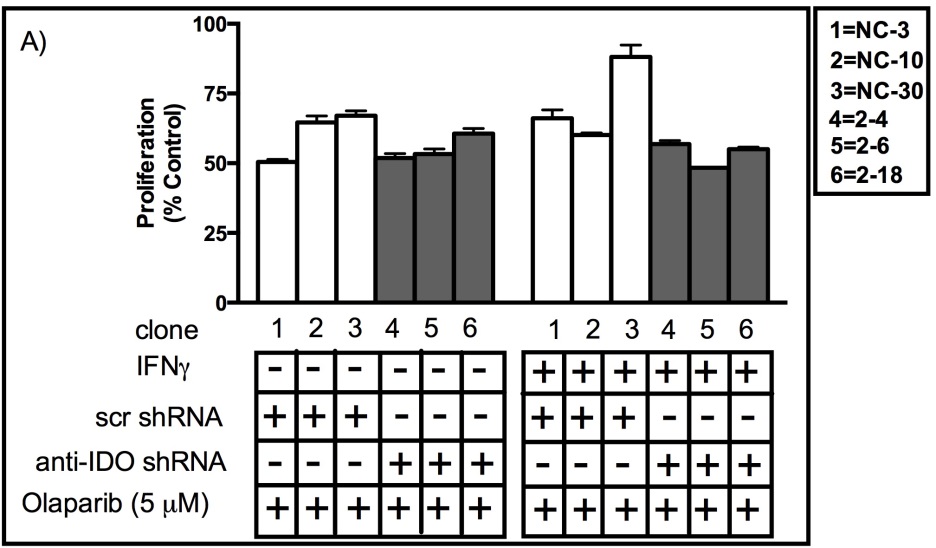


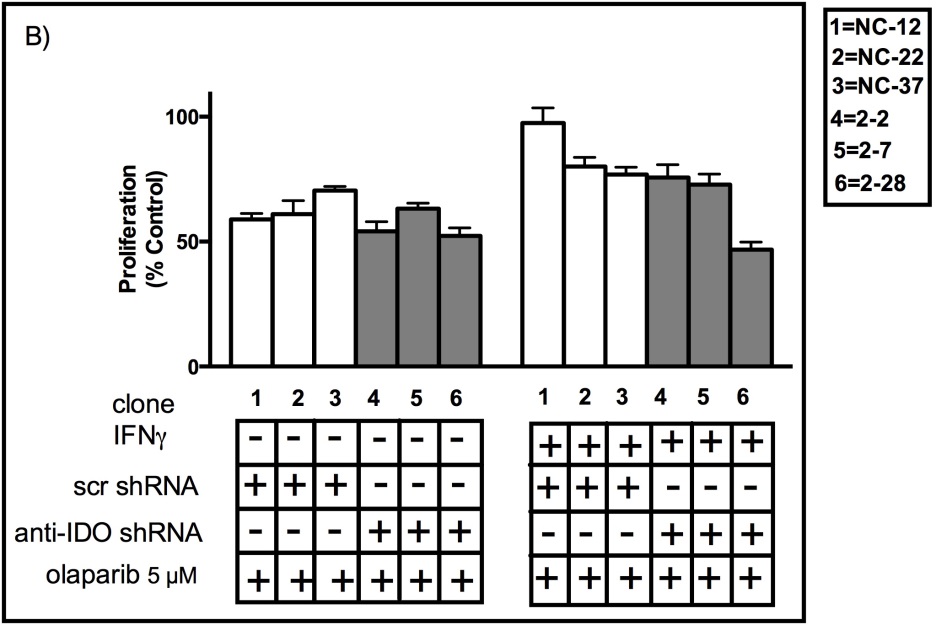


**Supplementary Figure 2:** (A) A549 and (B) HeLa clone sensitivity to high dose olaparib (5 μM) before and after IDO induction. **Panels A-B** present data for each of 6 individual clonal populations. Means derived from these results are presented in **Figure 3** of the paper. Results were obtained from each individual clonal cell population harboring scrambled control shRNA or anti-IDO shRNA, and each bar represents the mean of 3 replicates (*n*=3 for each replicate experiment) ± SD.

**
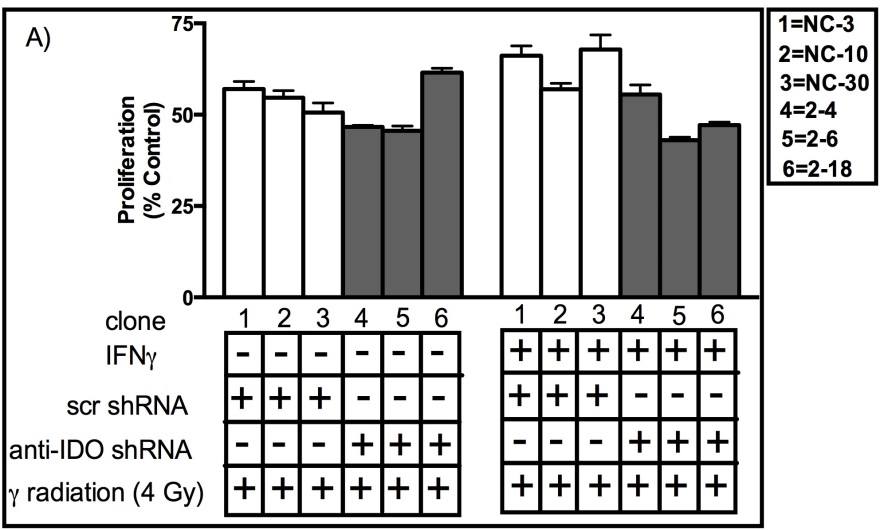
**

**
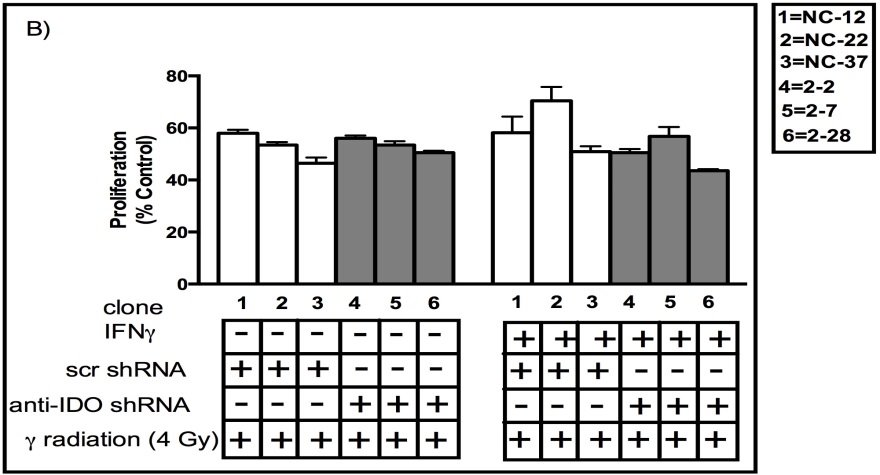
**

**Supplementary Figure 3: (A)** A549 and (B) HeLa clone sensitivity to γradiation (4 Gy) before and after IDO induction. Data for each of 6 individual clonal populations is presented. Means derived from these results are presented in **Figure 5** of the paper. Results were obtained from each individual clonal cell population with scrambled control shRNA or anti-IDO shRNA, and each bar represents the mean of 3 replicates (*n*=3 for each replicate experiment) ± SD.


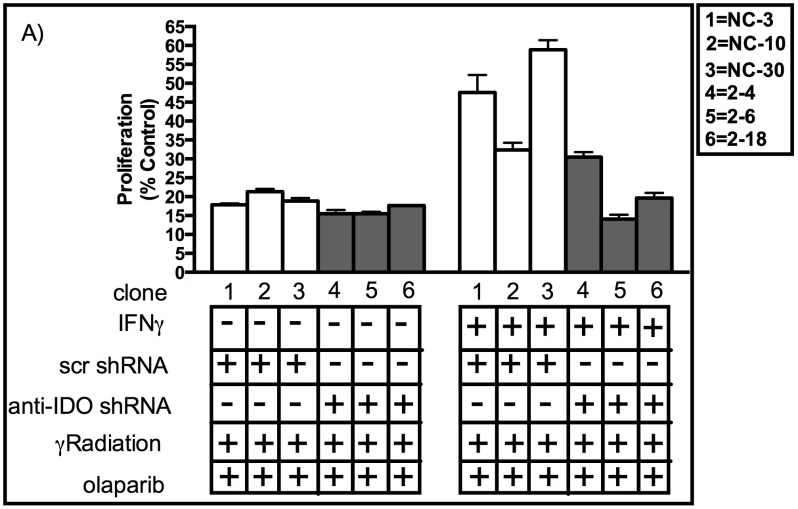


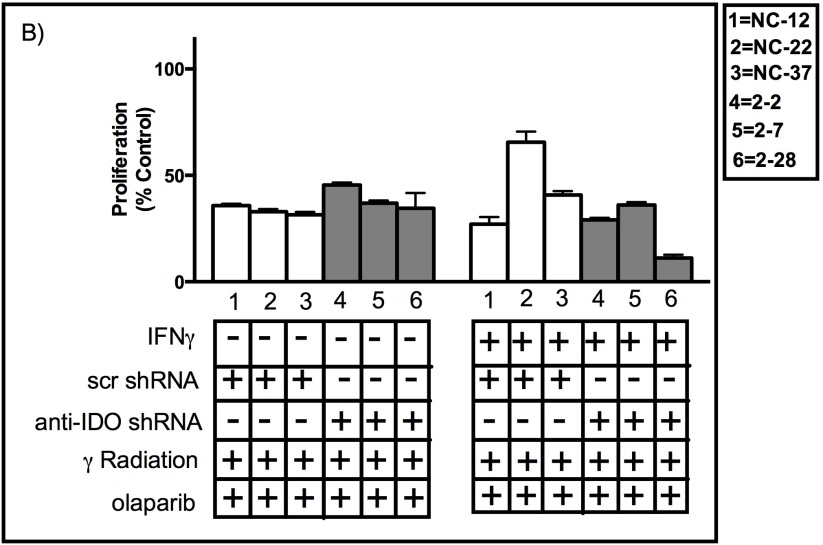


**Supplementary Figure 4:** (A) A549 and (B) HeLa clone sensitivity to combined γradiation (4 Gy) and olaparib (5 μM) treatment before and after IDO induction. Experimental repeat data for each of 6 individual clonal populations is presented. Means derived from these results are presented in **Figure 6** of the paper. Results were obtained from each individual clonal cell population with scrambled control shRNA or anti-IDO shRNA, and each bar represents the mean of 3 replicates (*n*=3 for each replicate experiment) ± SD.


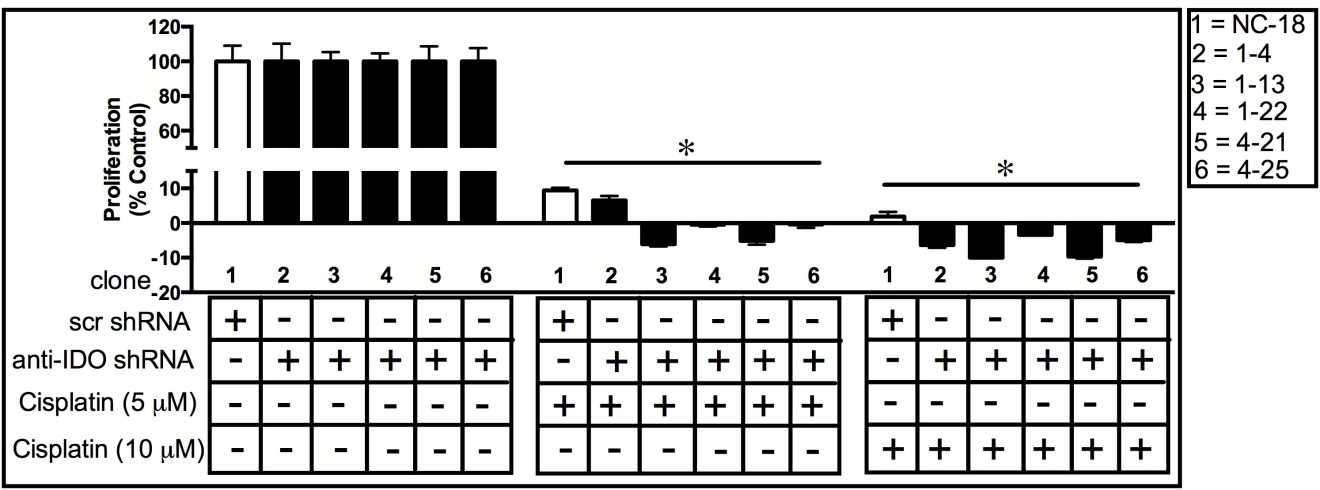


**Supplementary Figure 5:** H441 sensitivity to cisplatin (5 or 10 μM). Results were obtained from independent measurements of proliferation of 1 H441 clonal population harbouring control scrambled shRNA and 5 harboring anti-IDO shRNA. Bars represent the means of 3 independent measurements (*n*=3 for each measurement) ± SEM (**p*≤0.05).
